# Panel-based RNA fusion sequencing improves diagnostics of pediatric acute myeloid leukemia

**DOI:** 10.1038/s41375-023-02102-9

**Published:** 2023-12-12

**Authors:** Lina Marie Hoffmeister, Julia Suttorp, Christiane Walter, Evangelia Antoniou, Yvonne Lisa Behrens, Gudrun Göhring, Amani Awada, Nils von Neuhoff, Dirk Reinhardt, Markus Schneider

**Affiliations:** 1 Department of Pediatric Hematology and Oncology, University Children’s Hospital Essen, University of Duisburg-Essen, 45147 Essen, Germany; 2 Department of Human Genetics, Hannover Medical School, 30625 Hannover, Germany

**Keywords:** Genetics research, Cancer genomics

## Abstract

New methods like panel-based RNA fusion sequencing (RNA-FS) promise improved diagnostics in various malignancies. We here analyzed the impact of RNA-FS on the initial diagnostics of 241 cases with pediatric acute myeloid leukemia (AML). We show that, compared to classical cytogenetics (CCG), RNA-FS reliably detected risk-relevant fusion genes in pediatric AML. In addition, RNA-FS strongly improved the detection of cryptic fusion genes like *NUP98::NSD1*, *KMT2A::MLLT10* and *CBFA2T3::GLIS2* and thereby resulted in an improved risk stratification in 25 patients (10.4%). Validation of additionally detected non-risk-relevant high confidence fusion calls identified *PIM3::BRD1*, *C22orf34::BRD1*, *PSPC1::ZMYM2* and *ARHGAP26::NR3C1* as common genetic variants and *MYB::GATA1* as recurrent aberration, which we here describe in AML subtypes M0 and M7 for the first time. However, it failed to detect rare cytogenetically confirmed fusion events like *MNX1::ETV6* and other chromosome 12p-abnormalities. As add-on benefit, the proportion of patients for whom measurable residual disease (MRD) monitoring became possible was increased by RNA-FS from 44.4 to 75.5% as the information on the fusion transcripts’ sequence allowed the design of new MRD assays.

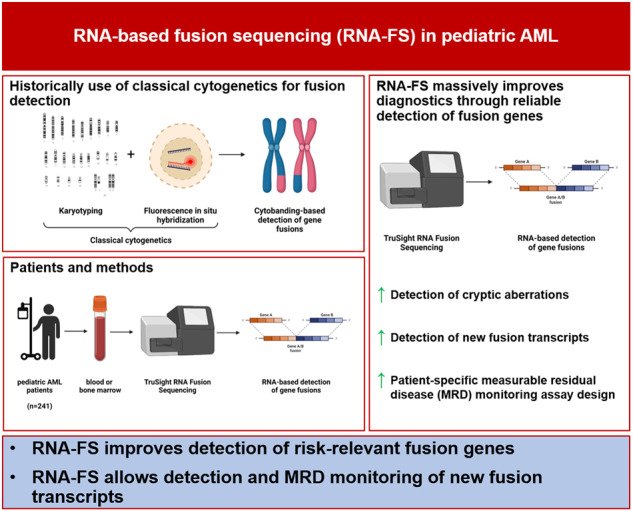

## Introduction

Pediatric acute myeloid leukemia (AML) is the second most common leukemia in children with an incidence of 0.7 per 100,000 patients annually in Germany [1]. In 75–80% of the cases various clonal chromosomal aberrations are detected [2–4]. Among these aberrations, translocations in pediatric AML are the most frequent often resulting in gene fusions. Regarding patient age, *KMT2A* fusions are the most common rearrangements in infants while CBF-fusions are more common in older children [4, 5]. As multiple genetic aberrations correlate with response after treatment, patients nowadays are stratified into defined standard-, intermediate- and high-risk groups. This adaption of treatment intensity by risk group improved the survival rates with 5-year probabilities for event-free survival (EFS) and overall survival (OS) from 41 ± 3% and 49 ± 3% in 1987 to 50 ± 2% and 76 ± 4%, respectively, in the most recent AML-BFM trial [1].

Evidently, a precise and rapid detection of the underlying genetic aberrations plays a major role when pediatric AML is diagnosed. Presently, the diagnostic tools applied comprise conventional and fluorescence in situ hybridization (FISH) karyotyping as well as next-generation sequencing (NGS). On a routine basis, karyotyping and FISH make up the largest part to detect the high number of aneuploidies and structural variants (SVs) occurring in pediatric AML and thus still form the gold standard when diagnosing AML [4, 6]. However, disadvantages in karyotyping and FISH still exist such as difficulties in identifying cryptic or balanced translocations by visual inspection of cytobands [4]. High throughput sequencing as a new promising method is coming up to detect those cryptic or balanced translocations [4, 6–9] but from a practical viewpoint, is still associated with high costs, the necessity of batching samples, time-consuming running and requirement of bioinformaticians for reporting of results [10].

Therefore, the routine diagnostic workflow at the initial diagnosis of the current pediatric AML-BFM 2017 registry has implemented panel-based RNA fusion sequencing (RNA-FS) as an additional tool to identify gene fusions missed by karyotyping and to investigate for an extended set of stratification relevant gene fusions. Besides aiming to improve the reliable detection of potentially missed fusion transcripts, newly identified fusion gene sequences may serve as targets for measurable residual disease (MRD) monitoring.

On this background, we analyzed material from 241 children consecutively diagnosed with de novo AML by RNA-FS. The results were compared to karyotyping, including FISH analysis, performed as part of the initial routine diagnostics. When fusion genes were detected, we assessed the applicability for MRD monitoring. To the best of our knowledge, we here present the first comprehensive analysis of fusion gene detection by high throughput sequencing in a large, unique cohort of 241 pediatric AML patients.

## Methods

### Study cohort

A total number of 258 pediatric patients (0–18 years) were diagnosed with AML in Germany from January 2019 to December 2021. Patients with Down syndrome and myelosarcoma were excluded. The RNA-FS and CCG data of 241 patients were available and included in this study. All patients were enrolled onto the AML-BFM 2017 registry in Germany (DRKS number: DRKS00013030). The protocol was approved by the institutional review board (University Hospital Essen, ethical vote number 17-7462-BO, 8 December 2017), conducted in accordance with the Declaration of Helsinki, and written informed consent was obtained from all patients and their legal guardians. Patients’ clinical characteristics are shown in Supplementary Table 1. Sampling of blood or bone marrow specimen was performed as part of the initial diagnostic routine.

### TruSight RNA-FS and fusion calling

RNA-FS was performed with RNA sampled at initial diagnosis of 251 patients using the TruSight RNA Fusion Panel (Illumina, San Diego, CA, USA) following the manufacturer’s recommendations. Only libraries with a concentration of >6.5 ng/µl and with a size of 250–300 bp were sequenced. Sequencing was performed on an Illumina MiSeqDX sequencer in research mode with 76 bp paired-end reads using the MiSeq Reagent Kit v3 (150-cycle). A minimum of two million reads per samples was required for analysis. The data analysis, including fusion calling, was performed on the MiSeqDX system using the RNA Fusion Analysis Module v2.0 of the local run manager (Illumina, San Diego, CA, USA) with default settings recommended by Illumina [11]. This module utilized the STAR Aligner [12] and the Manta algorithm [13]. In this study, we only considered high confidence fusion calls and recurrent, stratification-relevant fusion genes referred to the AML-BFM 2017 registry.

### Classical karyotyping and fluorescence in-situ hybridization

Classical cytogenetics (CCG) including karyotyping and fluorescence in-situ hybridization was performed on 248 patients at the Department of Human Genetics, Hannover Medical School, Hannover, Germany as part of the initial diagnostics as described previously [14, 15].

### Validation of detected gene fusions by RT-PCR, multiplex RT-PCR and OGM

Gene fusions detected by TruSight RNA-FS or CCG were validated by reverse transcriptase polymerase chain reaction (RT-PCR) [16], quantitative RT-PCR [17], multiplex RT-PCR (HemaVision®) [14] or optical genome mapping (OGM) [18]. Complementary DNA (cDNA) was synthesized by using SuperScript® VILO™ (Thermo Fisher Scientific, Carlsbad, CA, USA). For RT-PCR the ALLin™ Hot Start Taq Mastermix (highQu GmbH, Kraichtal, Germany) and for quantitative RT-PCR the TaqMan Universal PCR Master Mix (Thermo Fisher Scientific, Carlsbad, CA, USA) was used. Probes for qPCR were FAM/BHQ1 labled. For multiplex RT-PCR the HemaVision® Kit was used (HiSS Diagnostics GmbH, Freiburg im Breisgau, Germany). OGM was performed following manufacturers’ instructions using the SP Bone Marrow Aspirate DNA Isolation kit and the Direct Label and Stain (DLS) Kit (Bionano Genomics, San Diego, CA, USA). PCR-amplificates from RT-PCR were purified by gel electrophoresis followed by Sanger sequencing at a commercial laboratory (Microsynth Seqlab GmbH, Göttingen, Germany). All primers were designed using the SnapGene® software (www.snapgene.com; GSL Biotech LLC, San Diego, CA, USA) and PrimerBlast [19]. The method of validation was chosen on the basis of the use in diagnostics (qRT-PCR), the availability of sequence information (RT-PCR), the detectability in a multiplex approach (multiplex PCR) or the lack of applicability of the latter (OGM).

## Results

### Comparison of risk-relevant fusion genes detected by RNA-FS or CCG

**Fig. 1:**
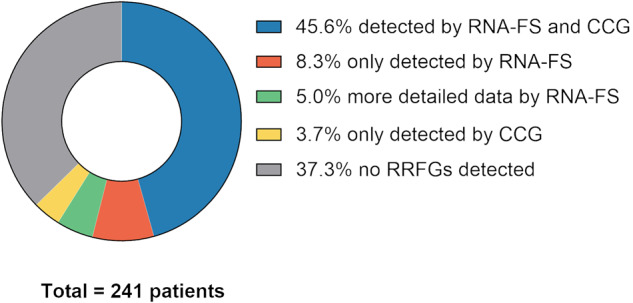
Distribution of risk-relevant fusion genes (RRFGs) (based on AML-BFM registry 2017) in pediatric AML patients (*n* **=** 241) regarding detection by RNA-FS, CCG or both. In 45.6% of patients (*n* = 110) RRFGs were detected by RNA-FS and CCG, in 8.3% (*n* = 20) by RNA-FS only and in 3.7% (*n* = 9) by CCG only. RNA-FS was able to describe *KMT2A*-r in more detail in 12 samples (5.0%). In 37.3% (*n* = 90) no RRFG was detected by either of the methods. CCG classical cytogenetics, *KMT2A*-r *KMT2A* rearrangement, RNA-FS panel-based RNA fusion sequencing, RRFGs risk-relevant fusion genes (excluding *MECOM*::*RPN1*).

In 241 samples from initial diagnosis of pediatric AML patients, the presence of risk-relevant fusion genes (RRFGs) as defined by the AML-BFM registry 2017 protocol was analyzed by CCG and RNA-FS in parallel and the obtained results were compared (Supplementary Table 2, Supplementary Fig. 1). The fusion genes of *MECOM::RPN1* resulting from inv(3)(q21q26) were excluded from the analysis as it results in the juxtaposition of the distal GATA2 enhancer and does not result in fusion transcripts of the coding sequence of both genes and thus reaches a natural limit of RNA-FS, which is achieved by the presence of fusions on the RNA-level [20–22]. In 83.0% (*n* = 200; Fig. 1) of the cases, CCG and RNA-FS detected identical results in respect to RRFGs, meaning the detection of the same RRFG (45.6%, *n* = 110) or the absence of RRFGs (37.3%, *n* = 90). RRFGs were detectable in 151 out of 241 patients (62.7%) by at least one of the latter methods (Fig. 1). Simultaneous presence of two RRFGs, namely *NUP98::NSD1* and *BCR::ABL1*, was found in one patient (case 44) resulting in stratification into the high-risk group. Therefore, 152 RRFGs were identified by either CCG or RNA-FS. A total of 148 of the 152 detected RRFGs (excluding *MECOM::RPN1*) were validated by an orthogonal detection assay (RT-PCR, RT-qPCR, multiplex RT-PCR or OGM). Two RRFGs could not be validated due to insufficient material for additional analyses (1.3%, *n* = 2; cases 54 and 97) and two RRFGs (1.3%, *n* = 2; cases 81 and 153) that were only identified by CCG could not be validated by neither of the latter methods. In detail, one of three 12p-abnormalities was not detected by OGM and its validation was not possible with other methods due to the lack of existing assays (case 153). Furthermore, one of 13 cases with t(10;11)(p12;q23) could not be validated by multiplex RT-PCR or by other methods due to lack of material (case 81).

**Fig. 2:**
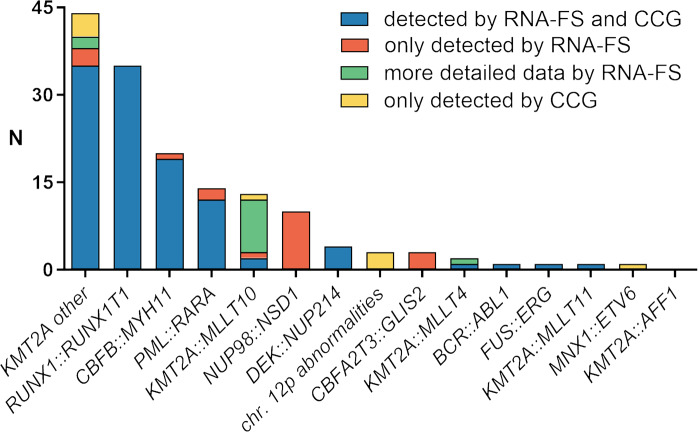
Frequency of risk-relevant fusion genes (RRFGs) (*n* = 152) of pediatric AML (based on AML-BFM registry 2017) detected by RNA-FS, CCG or both. CCG classical cytogenetics, RNA-FS panel-based RNA fusion sequencing, RRFGs risk-relevant fusion genes (excluding *MECOM::RPN1*).

Although most AML cases showed identical results regarding RRFGs, some could only be detected by either one of the two methods. In 8.3% (*n* = 20) of the cases RNA-FS detected RRFGs that were not detected by CCG. All cases with *NUP98::NSD1* (*n* = 10/10) and *CBFA2T3::GLIS2* (*n* = 3/3; cases 1, 130 and 131) were only identified using RNA-FS (Fig. 2). Rare cases of failure in fusion detection by CCG were observed concerning *CBFB::MYH11* (*n* = 1/20; case 133), *PML::RARA* (*n* = 2/14; cases 61 and 68) and other *KMT2A* rearrangements (*KMT2A*-r) (*n* = 3/44; cases 95, 106 and 124). In 12 cases (5.0%) both methods identified *KMT2A*-r as RRFGs, but only RNA-FS was able to identify the fusion partner gene of *KMT2A* and consequently to describe the RRFGs in more detail. These fusion genes comprised *KMT2A::MLLT10* (*n* = 9) but also *KMT2A::MLLT4* (*n* = 1; case 42), *KMT2A::USP2* (*n* = 1; case 114) and *KMT2A::SEPT6* (*n* = 1; case 110). Furthermore, in one case with *KMT2A::MLLT10* (*n* = 1; case 77) detected by RNA-FS no indication of *KMT2A*-r could be found by means of CCG. On the other hand, in 3.7% (*n* = 9) of cases RRFGs were only detected by CCG. In particular, abnormalities in chromosome 12p (*n* = 3; cases 151–153) and fusion gene *MNX1::ETV6* resultig from t(7;12)(q36;p13) (*n* = 1; case 37) were detected by CCG only. In addition, RNA-FS failed to detect four cases with *KMT2A*-r (*n* = 4/44; cases 115–118) which were not further specified by CCG. On closer inspection, the four cases with undetected *KMT2A*-r in RNA-FS showed aberrations involving *KMT2A*, which were, however, considered imprecise by the Manta algorithm. Furthermore, one case with *KMT2A::MLLT10* resulting from t(10;11)(p12;q23) (*n* = 1/13; case 81) was not detected by RNA-FS, and as of note, could not be validated by multiplex RT-PCR because the remaining material was not sufficient for further validation. But also in this case an aberration of *KMT2A* was considered imprecise by the Manta algorithm of RNA-FS.

Only considering the detection of RRFGs for the stratification into the three established risk categories, a total of 25 of 241 patients (10.4%) benefit from the additional use of RNA-FS. These 25 patients would all have been stratified into the intermediate-risk group by applying CCG alone, but fusion transcripts that led to aberrant risk stratification were identified by RNA-FS resulting in a shift of all but one patient (*n* = 24) into high-risk group. The remaining patient (case 133) could be stratified into the standard-risk group as RNA-FS identified an inv(16)(p13q22) leading to the expression of *CBFB::MYH11* which was not detected by CCG.

### Detection of additional fusion transcripts by RNA-FS

**Fig. 3:**
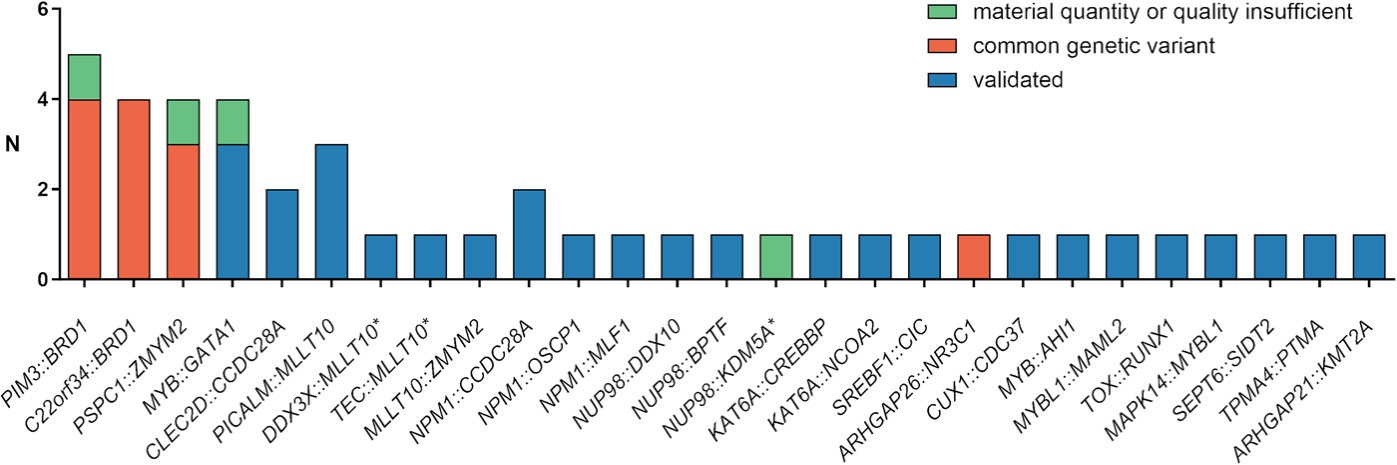
RNA-FS identified common genetic variants and recurrent non-RRFGs in a cohort of 241 pediatric AML patients. RNA-FS panel-based RNA fusion sequencing, non-RRFGs non-risk-relevant fusion genes, * reciprocal fusion transcript in same patient detected.

In 36 of 241 patients (14.9%; Supplementary Fig. 1), analyzed by RNA-FS, non-risk relevant fusion genes (non-RRFGs) were detected (Fig. 3) and in 25 out of these cases (69.4%) no co-occuring RRFGs was detected. In total, RNA-FS identified 27 different non-RRFGs (excluding reciprocal non-RRFGs), with some occurring recurrently and also in parallel with other non-RRFGs or RRFGs within the same patient. Excluding reciprocal non-RRFGs this resulted in the detection of a total of 45 non-RRFGs in all patient samples (mean non-RRFGs per patient: 1.2, min: 1, max: 3). Validation experiments by RT-PCR, gel electrophoresis and Sanger sequencing revealed *PIM3::BRD1* (*n* = 4; cases 21, 67, 109 and 120), *C22orf34::BRD1* (*n* = 4; cases 20, 23, 131 and 203), *PSPC1::ZMYM2* (*n* = 3; cases 67, 204, 205) and *ARHGAP26::NR3C1* (*n* = 1; case 202) as common genetic variants being detected in pooled RNA of five healthy donors as well as in leukemic blasts (Supplementary Fig. 2). All other fusion genes with enough RNA for validation were confirmed (*n* = 28) (Supplementary Fig. 3).

Some fusion genes such as *MYB::GATA1* (*n* = 4; cases 208, 209, 210 and 220), *PICALM::MLLT10* (*n* = 3; cases 152, 218 and 241)*, CLEC2D::CCDC28A* (*n* = 2; cases 212 and 213) and *NPM1::CCDC28A* (*n* = 2; cases 212 and 213) were detected recurrently (Supplementary Table 2). *CLEC2D::CCDC28A* and *NPM1::CCDC28A* occurred simultaneously in the same patients (cases 212 and 213) and were exclusively detected in female patients with morphologic subtype M4 of infant AML. One patient is still alive (case 213), however, the other died 1.3 years after diagnosis due to relapse of the disease (case 212). *PICALM::MLLT10* was detected in three adolescent patients with morphologic subtypes M0 or M1. Two of them died, one early before the start of induction treatment (case 218) and the other one 1.4 years after diagnosis due to relapse (case 152) while the third patient is still in remission (case 241). *MYB::GATA1* fusion genes were detected in four infant patients (cases 220, 208, 209 and 210) with varying morphologic subtypes (M0, M6, 2x M7) and gender (3x male, 1x female) (Supplementary Table 2). Two of these four patients exhibiting subtype FAB M0 and M6, died one month (case 209) and 14 months (case 210) after AML diagnosis, due to multiorgan failure or relapse, respectively.

Genes, such as *NPM1*, *NUP98, KAT6A* and *MLLT10* were recurrently involved in fusion events. *NPM1*-rearrangements (cases 212–215), such as *NPM1::OSCP1* (*n* = 1; case 215), *NPM1::MLF1* (*n* = 1; case 214) and *NPM1::CCDC28A* (*n* = 2; cases 212 and 213) exclusively occurred in AML subtype M4 (*n* = 4/4) and preferably in infant patients (*n* = 3/4; cases 212, 213 and 215). In addition to the well-known aberration *NUP98::NSD1*, *NUP98* was rearranged with *BPTF* (*n* = 1; case 216), *DDX10* (*n* = 1; case 217) or *KDM5A* (*n* = 1; case 219). Patients with *NUP98*-rearrangements showed morphologic subtype M7 (*n* = 2/3; cases 216 and 219) or M2 (*n* = 1/3; case 217) and were diagnosed at infant (*n* = 1/3; case 216) or early childhood age (*n* = 2/3; cases 217 and 219). *KAT6A* was fused to *CREBBP* (*n* = 1; case 206) or *NCOA2* (*n* = 1; case 207). Patients with *KAT6A*-rearrangements showed no matches in age, gender or morphologic subtype. Besides the well known rearrangements of *MLLT10* with *KMT2A* or *PICALM*, *MLLT10* fused to *DDX3X* (*n* = 1; case 240), *TEC* (*n* = 1; case 223) and *ZMYM2* (*n* = 1; case 70). The affected cases were associated with morphologic subtype M5 (*n* = 3/3; cases 70, 223 and 240) and younger age at diagnosis (0.9–4.6 years).

Overall, considering RRFGs and non-RRFGs, RNA-FS detected blast-specific fusion transcripts in 68.5% (*n* = 165) of patients of which 7.9% (*n* = 19) harbored only non-RRFGs.

### TruSight RNA-FS enables MRD monitoring in a greater proportion of patients

**Fig. 4:**
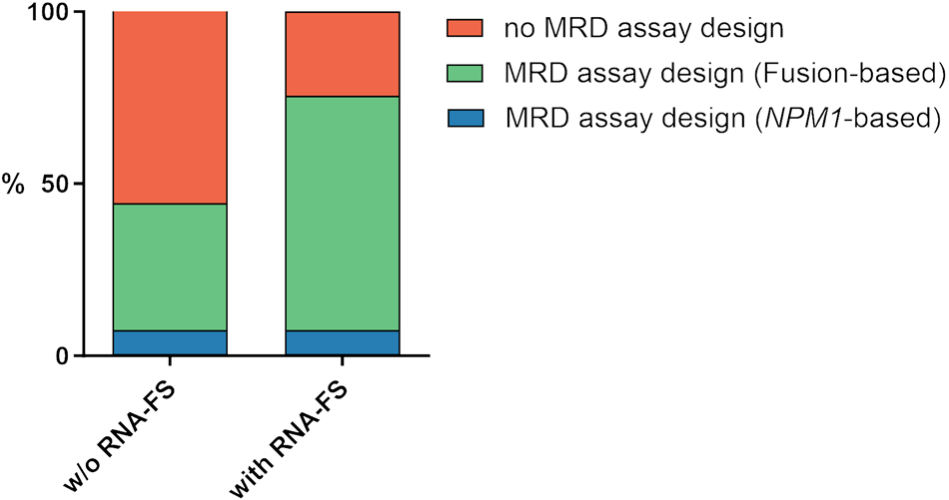
Impact of RNA-FS on MRD monitoring assay design. CCG, *NPM1*-mutational status and sequence information obtained by RNA-FS, enabled patient-specific MRD monitoring assay design in 75.5% of pediatric AML patients (*n* = 241). Without RNA-FS, MRD monitoring was possible for 44.4% of patients due to previously published assays. MRD measurable residual disease, RNA-FS panel-based RNA fusion sequencing, w/o without.

In the past, our laboratory has been able to monitor MRD in a total of 44.4% of patients during induction chemotherapy using previously published quantitative RT-PCR assays [23, 24]. Of these, 7.5% of the patients carried *NPM1* mutations detected by NGS and in 36.9% of the patients a *CBFB::MYH11*, *KMT2A::MLLT3*, *PML::RARA* or *RUNX1::RUNX1T1* rearrangement was identified by cytogenetic analyses. By additionally using RNA-FS and thus obtaining the sequence information of detected fusion genes we were able to identify fusion breakpoints on exon-level enabling the design of MRD monitoring assays (Supplementary Table 3). This increased the proportion of cases for which fusion-based MRD monitoring was possible to 68.0% of all patients (excluding common genetic variants; *n* = 164/241). Overall, using the DNA and RNA sequencing approaches to select molecular markers for MRD monitoring a total of 75.5% of patients could be monitored (Fig. 4).

Altogether, in our cohort we detected 36 fusion genes with 56 different fusion breakpoints on exon-level by RNA-FS suitable for MRD monitoring. This included recurrent gene fusions with stable fusion breakpoints, involving always the same exons, such as *RUNX1::RUNX1T1* (*n* = 35; 1 breakpoint), *NUP98::NSD1* (*n* = 10; 1 breakpoint), *DEK::NUP214* (*n* = 4; 1 breakpoint; cases 38–41) or *MYB::GATA1* (*n* = 4; 1 breakpoint; cases 208–210 and 220). In addition, highly divergent fusion genes with variable breakpoints and breakpoint regions, e.g. *CBFB::MYH11* (*n* = 20; 3 breakpoints), *CBFA2T3::GLIS2* (*n* = 3; 2 breakpoints; cases 1, 130 and 131) or *PICALM::MLLT10* (*n* = 3; 3 breakpoints; cases 152, 218 and 241) were detected. However, fusion transcripts involving *KMT2A* were the most variable ones. Overall, we found 57 patients having a fusion gene involving *KMT2A*. A total of 13 different fusion partner genes with 28 different fusion breakpoints were identified. Hereby, *KMT2A::MLLT10* was the most divergent fusion gene showing seven different breakpoints in 12 patients.

## Discussion

In this study, we aimed to examine the clinical impact of fusion gene detection in pediatric AML by the high resolution methodology RNA-FS, especially for risk-stratification and patient management during treatment. Therefore, 241 patients were analyzed with RNA-FS and CCG in parallel.

Comparing the panel-based fusion detection (RNA-FS) and CCG with regard to the detection of RRFGs, concordant results were found in the majority of cases (83.0%). In 62.7% of the patients at least one RRFG was detected by either one or both of the methods applied, whereby we were able to describe one rare case with two RRFGs, namely *NUP98::NSD1* and *BCR::ABL1*. Discordant results in terms of RRFG detection via RNA-FS and CCG were shown in 17.0% of cases. In individual cases, RRFGs reported by CCG were not called in RNA-FS by using the described procedure for fusion calling. However, it seems that in most of the cases these discrepancies are rather to be regarded as exceptions of individual cases than methodological failures in the detection of specific fusion transcripts. For example, aberrations involving the *KMT2A* gene were considered as imprecise by the Manta algorithm in five cases and thus not called. Furthermore, systematic failure of RNA-FS based on the detection of RRFGs was observed if 12p-abnormalities (*n* = 3) were present and also in one case with *MNX1::ETV6* (*n* = 1). As both aberrations are rarely detected cytogenetically in the presented cohort this finding should be validated in a bigger cohort. A possible explanation for the failed detection of *MNX1::ETV6* aberrations via RNA-FS might be that the fusion was only detected in 50% of cases on a transcript level but never on protein level. Chromosomal aberration rather influences the mRNA-expression level of *MNX1* instead of resulting in a functional fusion transcript and translation into a protein [25–27]. Panel-based fusion detection therefore might not be sufficient for the detection of this aberration. Perhaps it might be helpful to additionally generate DNA-based fusion detection or gene expression data to identify those kinds of alterations.

CCG systematically failed to detect *NUP98::NSD1* and *CBFA2T3::GLIS2*. Additionally, *MLLT10* was often not identified as a partner gene in *KMT2A::MLLT10* fusions. As all the latter are cryptic aberrations they need to be identified by other molecular methods [28–30] and at least suitable FISH probes should be used. But especially for *KMT2A::MLLT10* which is known to comprise multiple exons of *KMT2A* and *MLLT10* [31] that might be involved in fusions, high throughput methods like RNA-FS will be superior to FISH probes and PCR-based methods. As the fusion of *KMT2A* with different fusion partner genes shows different effect on the outcome [32, 33], the sole use of classical karyotyping with *KMT2A* split apart probes without further investigation with other methods is not sufficient for the initial characterization of pediatric AML.

Though RNA-FS improves the detection of RRFG, for a complete risk stratification in an optimized AML study, it is necessary to also cover other genetic aberrations that do not lead to the expression of a fusion transcript. These include for example inv(3)(q21q26) (*MECOM::RPN1*), which leads to an enhancer rearrangement and thus in *MECOM* overexpression but does not result in the expression of a fusion transcript [20–22]. Furthermore, *MNX1::ETV6* leads to a fusion transcript in only half of the affected patients and was not observed to be translated into a fusion protein yet [25–27], which is in concordance with our data. Instead *MNX1::ETV6* is known to result in ectopic *MNX1* expression. Also, numeric aberrations, like monosomy seven or trisomy eight, that are typically detected by CCG, need to be identified. This requires DNA-based methods for the detection of structural and numerical aberrations as well as the identification of gene mutations (indel, missense, nonsense). RNA-FS therefore improves the diagnostics with regard to the detection of RRFGs, but cannot replace CCG entirely. The substantial benefit for patient care of additionally performed RNA-FS was highlighted in our study through refinements in risk stratification. Risk-dependend tailoring of treatment intensity became possible based on RNA-FS for a significant proportion of patients (10.4% of our cohort) who all were categorized by the current AML-BFM protocol criteria as intermediate-risk based on CCG alone. As the majority of AML relapses historically is observed in the intermediate-risk group and there is still much debate about the definition and post-remission therapy of the patients belonging to this group, RNA-FS will contribute to solving these issues [34, 35].

In 14.9% (*n* = 36) of the patients, analyzed with RNA-FS, non-RRFGs were detected. In only a quarter of them a co-occurring RRFG was detected. This goes in line with observations of Bolouri et al. [5], reporting 68–90% of the patients harboring structural aberrations depending on the age group. Among the patients harboring at least one non-RRFG, an average of 1.2 non-RRFGs was detected (range 1–3). In general, all newly identified fusion transcripts should be validated by orthogonal methods and in addition their absence in healthy samples should be confirmed. Our analysis of non-RRFGs highlighted potential common genetic variants that also occur in the healthy population and therefore functional analyses are needed to rule out their pathogenicity. But in any case, investigators should be cautious about reporting these variants, especially when a fusion transcript is called by the algorithm, where both affected genes are located close by on the same chromosome. For instance, the genes involved in the common genetics varaints, identified in this study, were seperated by less than 200 kb. Furthermore, the analysis showed many fusion transcripts that occur only once, which underlines the heterogeneity of AML [36]. Evidently this underscores the potential of the method to discover new variants, however, in larger cohorts, it remains to be verified whether these are recurrent aberrations. The reliable detection of all fusion events will potentially result in the identification of new risk factors with prognostic significance transforming a nowadays non-RRFG into a RRFG in future trials.

Nevertheless, we have shown that some genes are more frequently involved in fusions albeit with various fusion partner genes (*MLLT10*, *NPM1*, *NUP98*, *KAT6A*). *MLLT10* is known to be frequently involved in fusion genes. Predominantly, *MLLT10* fuses to *KMT2A* or *PICALM*, but other less frequent partner genes were reported as well. *MLLT10*-rearrangements are associated with adverse outcomes regardless of the involved partner gene [37]. *NUP98*-rearrangments were recently characterized by Bertrums et al. [38], who were able to show two major groups, *NUP98::NSD1* and *NUP98::KDM5A*, and a minor group of *NUP98*-X fusions, which include various partner genes. Bertrums et al. [38] recommends to treat *NUP98*-rearranged AML in high-risk arms of trials regardless of the fusion partner gene due to the comparable molecular and clinical features within this subgroup. Also groups of *KAT6A*- and *NPM1*-fusions are known in adult acute myeloid leukemia [39–41]. We were able to detect the rare but recurrent fusion transcripts of *PICALM::MLLT10* (*n* = 3) in our cohort, which correlated with adolescence age, confirming previously published data [5]. Furthermore, we could detect four cases with the recurrent aberration *MYB::GATA1*. Previously published case reports point to a correlation with infant age and basophilic subtype or FAB M5 or FAB M6 [42–47]. In addition to these reports, we describe here for the first time an occurrence of *MYB::GATA1* in pediatric AML patients with a morphological FAB subtype M0 and M7. The functional impact of the aberration and the impact on survival need to be analyzed in a larger cohort.

RNA-FS allows the identification of so far unknown fusion transcripts. By providing sequence data of the patient-specific fusion breakpoints, it was possible to design RT-qPCR-based patient-specific molecular MRD monitoring assays. This has enabled us to increase the proportion of patients for whom we could offer MRD monitoring compared to the situation when we only used previously published MRD assays for the most recurrent gene fusions like *RUNX1*::*RUNX1T1*, *CBFB*::*MYH11*, *KMT2A*::*MLLT3* and *PML*::*RARA* [23, 24]. Evidently, this is associated with a great benefit for identification of treatment failure and thus steering the treatment of these patients. However, it will be of importance to consider the suitability of the newly identified patient-specific fusion transcripts as MRD markers. Therefore, these new assays have to be properly validated and the sensitivity limit determined. In general, it cannot be assumed that the presence of each fusion transcript is genetically stable during the disease and thus is representative for the persistence or reoccurrence of leukemic clones [48]. For molecular MRD monitoring of *RUNX1::RUNX1T1* fusion transcripts a prognostic significance was shown after first and second induction in pediatric AML [49]. Thus, assessment of the therapeutic response in the context of molecular MRD diagnostics is a crucial part in the current AIEOP-BFM AML 2020 trial protocol (EudraCT: 2020‐005634‐15) highlighting the clinical importance of MRD monitoring in the future.

Overall, our data underscore that RNA-FS offers a suitable method to substantially improve pediatric AML diagnostics by complementing existing methods. In particular, via improved risk-stratification patients will directly benefit from tailored therapy in a greater extent by the better detection of cytogenetically cryptic fusion genes. In addition, discovering new fusion transcripts and studying their impact on outcome will also contribute to a genetically-based improved risk stratification. Last but not least, the proportion of patients extended by 30% for whom MRD monitoring becomes applicable will profit from precise steering of AML treatment.

### Supplementary information

Supplemental Table 1 + Figure 1-3

Supplemental Table S2+S3

## Data Availability

For original data and primer sequences, please contact LinaMarie.Hoffmeister@uk-essen.de or Markus.Schneider@uk-essen.de. The sequencing data are deposited in the Sequence Read Archive (SRA) (SUB13582559).
