# Supplementary material for: Panel-based RNA fusion sequencing improves diagnostics of pediatric acute myeloid leukemia
Source: Leukemia. 2023 Dec 12;38(3):538–44. doi: 10.1038/s41375-023-02102-9 (PMC10912021; doi:10.1038/s41375-023-02102-9)
Supplement: Supplementary file 1 — Supplemental Table 1 + Figure 1-3 [file 41375_2023_2102_MOESM1_ESM.pdf]

Supplemental Table 1. Clinical characteristics of pediatric AML patients analyzed by RNA-FS, CCG or both

		All patients	Analyzed with RNA-FS	Analyzed with CCG	Analyzed with both methods
number (analyzed)		258	251	248	241
age at diagnosis	median	8.7	9.1	9.0	9.2
	range min	0.0	0.0	0.0	0.0
	range max	18.0	18.0	18.0	18.0
gender	male	137	132	134	129
	female	121	119	114	112
WBC count, x10^3/µl	median	21.1	23.48	21.4	23.5
	range min	0.6	0.6	0.6	0.6
	range max	700.4	700.4	700.4	700.4
Hemoglobin level, g/dl	median	7.95	7.9	8	8
	range min	1.7	1.7	1.7	1.7
	range max	17.7	17.7	17.7	17.7
Platelet count, x10^3/µl	median	58	57	57.5	57
	range min	4	4	4	4
	range max	393	393	393	393
Risk groups	standard risk	67	66	65	64
	intermediate risk	102	98	100	96
	high risk	64	63	59	58
	APL standard risk	9	9	8	8
	APL high risk	5	5	5	5
	no data	11	10	11	10
morphologic subtype (FAB classification)	M0	5	5	5	5
	M1	33	33	33	33
	M2	60	58	58	56
	M3	15	15	14	14
	M4	45	45	44	44
	M4 eo	21	21	20	20
	M5	55	53	52	50
	M6	3	2	3	2
	M7	18	16	16	14
	no data	3	3	3	3

CCG = classical cytogenetics
FAB = French-American- British
RNA-FS = RNA-based fusion sequencing
WBC = white blood cell count

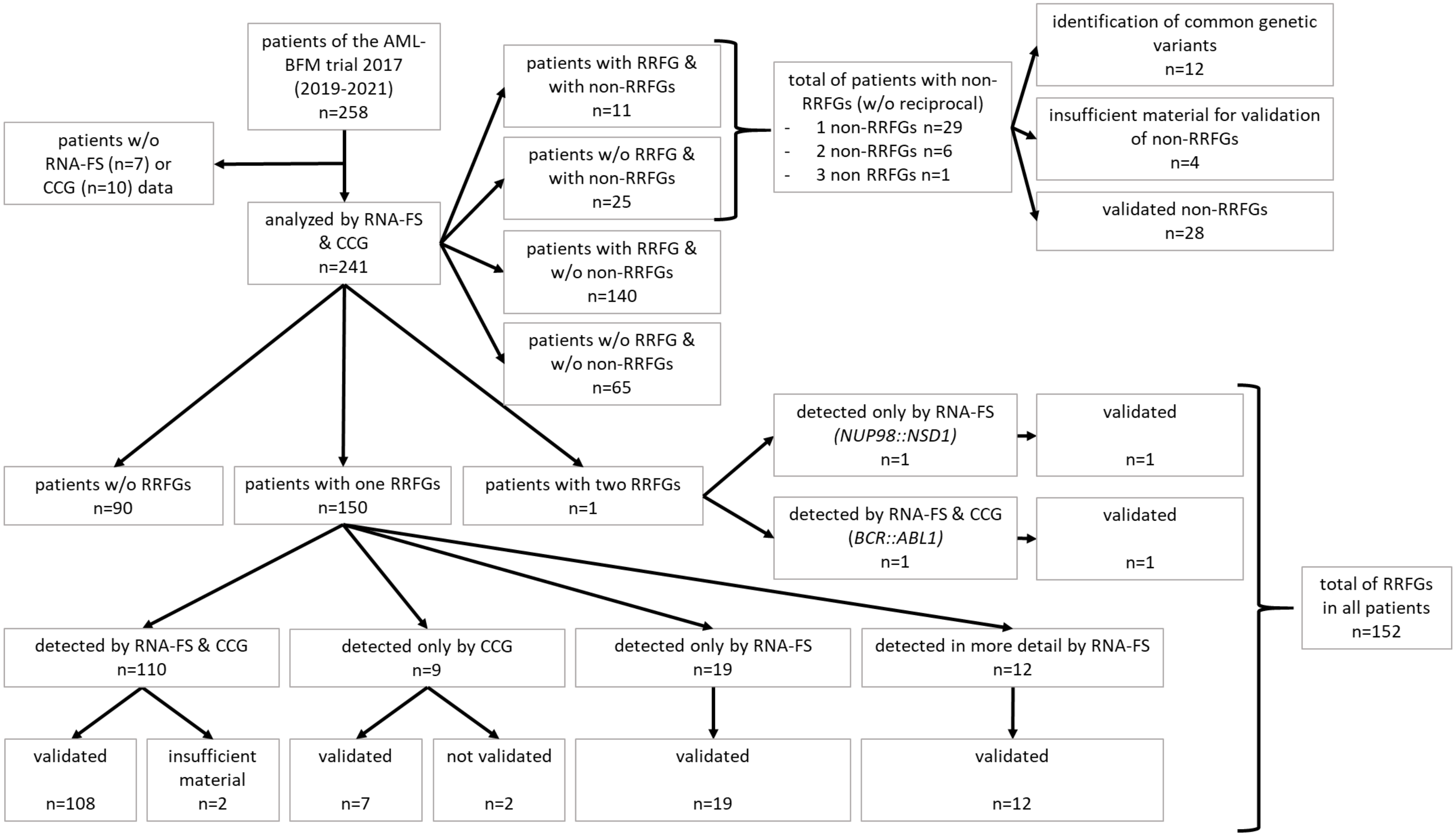

Supplemental Figure 1. Consort Chart about the study. Abbreviations: CCG = classical cytogenetics, RNA-FS = panel-based RNA-Fusion Sequencing, RRFG = risk-relevant fusion gene, w/o = without.

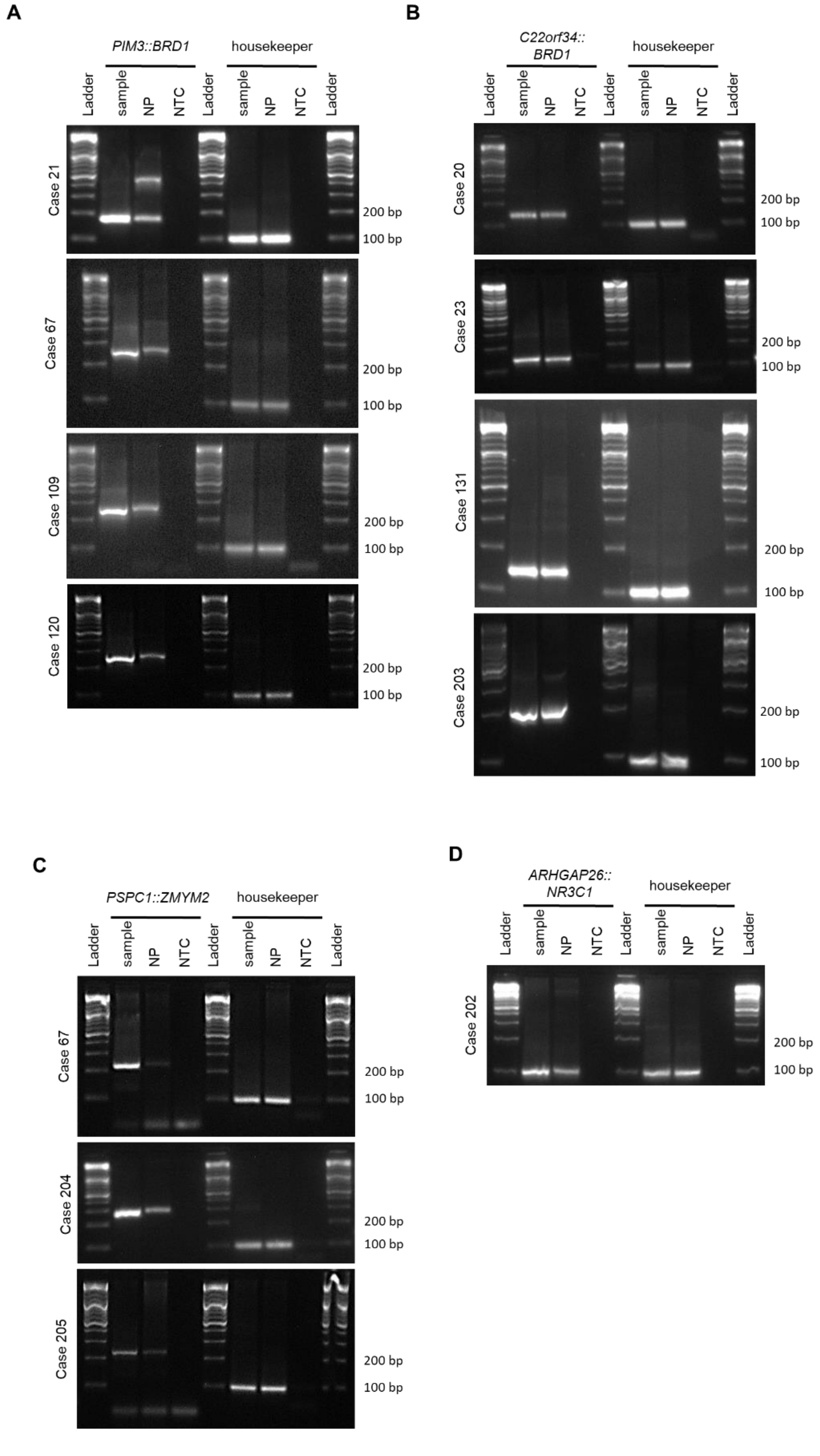

Supplemental Figure 2. Validation of common genetic variants. In all cases, fusion-spanning PCR was performed with forward und reverse primers on patients' cDNA collected at the day of initial diagnosis and on pooled cDNA of five healthy donors generating a PCR product with the expected length. In every case, the healthy control cDNA was positive, too. The no template control was negative in every case. (A) Validation of *PIM3::BRD1* in cases 21, 67, 109, 120 via RT-PCR. Fusion spanning RT-PCR generated a PCR-product with the expected length of 179 bp or 263 bp. (B) Validation of *C22orf34::BRD1* in cases 20, 23, 131, 203 via RT-PCR. Fusion-spanning PCR generated a product with the expected length of 140 bp or 219 bp. (C) Validation of *PSPC1::ZMYM2* in cases 67, 204, 205 via RT-PCR. Fusion-spanning PCR generated a product with the expected length of 236 bp. (D) Validation of *ARHGAP26::NR3C1* in case 202 via RT-PCR. Fusion-spanning PCR generated a product with the expected length of 104 bp. Ladder: GeneRuler DNA Ladder Mix. Abbreviations: NP = healthy control cDNA, NTC = no template control, PCR = polymerase chain reaction.

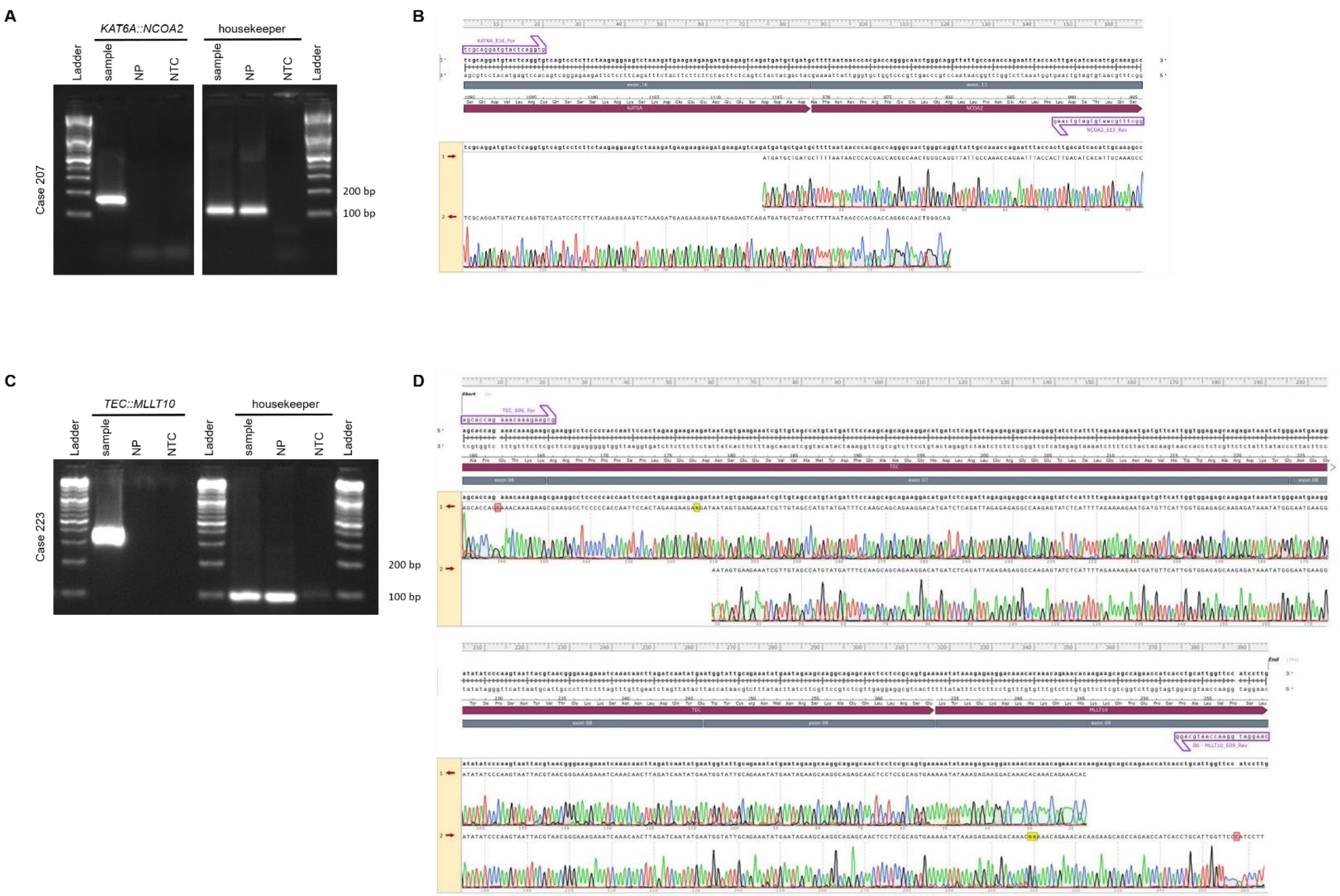

Supplemental Figure 3. Examples for validation of non-RRFG by qRT-PCR and Sanger Sequencing. Fusion-spanning PCR was performed with forward und reverse primers on patients' cDNA collected at the day of initial diagnosis generating a PCR product with the expected length. Healthy control cDNA, consisting of pooled cDNA of five healthy donors, was negative for non-RRFGs but positive for housekeeper genes. The no template control was negative in every case. (A) Validation of *KAT6A::NCOA2* in case 207 via RT-PCR. Fusion-spanning RT-PCR generated a PCR-product with the expected length of 166 bp. (B) Sanger Sequencing confirmed the presence of *KAT6A::NCOA2* in case 207. (C) Validation of *TEC::MLLT10* in case 223 via RT-PCR. Fusion-spanning PCR generated a product with the expected length of 394 bp. (D) Sanger Sequencing confirmed the presence of *TEC::MLLT10* in case 223. Ladder: GeneRuler DNA Ladder Mix. Abbreviations: NP = healthy control cDNA, NTC = no template control, PCR = polymerase chain reaction.
